# Fabrication and Characterization of Ice Templated Membrane Supports from Portland Cement

**DOI:** 10.3390/membranes10050093

**Published:** 2020-05-09

**Authors:** Amanmyrat Abdullayev, Paul H. Kamm, Maged F. Bekheet, Aleksander Gurlo

**Affiliations:** 1 Fachgebiet Keramische Werkstoffe/Chair of Advanced Ceramic Materials, Institute of Materials Science and Technology, Technische Universität Berlin, 10623 Berlin, Germany; Maged.Bekheet@ceramics.tu-berlin.de (M.F.B.); Gurlo@ceramics.tu-berlin.de (A.G.); 2 Institute of Applied Materials, Helmholtz-Zentrum Berlin für Materialien und Energie, Hahn-Meitner-Platz 1, 14109 Berlin, Germany; paul.kamm@helmholtz-berlin.de

**Keywords:** freeze casting, cement, membrane, sintering-free, water flux

## Abstract

Porous ceramic membranes for aqueous microfiltration and ultrafiltration processes suffer from the high-costs of material and processing. The latter is mainly due to the high-temperature sintering step. In this work, cement-based membrane supports from ultrafine Portland cement are studied as a low-cost alternative to traditional oxidic ceramic supports. An environmentally friendly freeze-casting fabrication route is applied for the fabrication of porous membrane supports. Cement membrane supports are becoming mechanically stabile after hydration reaction of cement with water, which does not require any high-temperature sintering step as in a conventional ceramic membrane fabrication process. This fabrication route, which is sintering-free, decreases the cost and environmental impact of the membrane fabrication process by eliminating extra energy consumption step during sintering. The Archimedes method, scanning electron microscopy (SEM), micro-computed tomographic (µCT), and mercury porosimetry characterize the membrane supports in respect to open porosity, pore size distribution, morphology, and connectivity. The flexural strength of the 3 mm thick membranes is in the range from 1 to 6 MPa, as obtained by the ring-on-ring tests. The obtained membrane supports possess porosity in the range between 48 and 73% depending on fabrication conditions (cooling rate and the solid content, as determined by Archimedes method enabling water flux in the range between 79 and 180 L/(h·m^2^) at 0.5 bar transmembrane pressure difference and 3 mm membrane thickness.

## 1. Introduction

Membranes have found a broad range of applications in chemical, biological, food, pharmaceutical, and energy industries due to the simple construction and energy efficiency of membrane technology when compared to other separation technologies, such as distillation [1]. Most commercially available membranes are made from organic polymers, as these offer the advantages of low production costs coupled with readily tunable porosity. However, in recent years, inorganic ceramic membranes have also attracted increasing attention due to their robustness, lifecycle, and energy efficiency. Moreover, ceramic membranes have good stability in harsh environmental conditions, e.g., bacterial attack, acidic or alkaline environment, and high temperature [2]. However, in contrast to polymeric membranes, ceramic membranes are more expensive due to their high production costs, which result from their costly raw materials (alumina, zirconia, titania) and high processing temperatures [3]. Most recently, the trend in research has been towards reducing the production costs of ceramic membranes by using low-cost raw materials, low processing temperature, and/or simple processing routes [4,5,6]. For instance, several readily available raw materials, such as kaolin, fly ash, and rice husk ash-based silica, have been extensively used in the production of ceramic membranes [7,8,9]. However, these low-cost materials still require high-temperature sintering, i.e. >1000 °C, despite the utilization of various sintering aids or processes like co-sintering [10,11]. To further decrease the production costs and environmental effects, the development of low-temperature or sintering-free membrane fabrication routes are still required. A comprehensive survey of preparing ceramic membranes from low-cost materials, such as clays, zeolites, apatite, bauxite, fly ash, rice husk ash, and cement is provided in our recently published review [6]. The fabrication of ceramic membranes from ashes, clays, apatite, and quartz sand needs relatively low sintering temperatures in comparison with alumina, zirconia, and titania, according to our survey. In contrast, cement-based membranes do not require sintering, and their fabrication is considered to be among the more cost-effective and environmentally friendly approaches to these systems.

Cement is a promising raw material for the production of inorganic membranes, not only because of its low cost, but also due to its good hardness by time after reacting with water, i.e., sintering-free route [12]. Portland cement has been used as a primary constituent of concrete for many decades and it is a very well-studied material [13]. The main constituents of Portland cement are tricalcium silicate and dicalcium silicate, which produce calcium silicate hydrate (C–S–H) by reaction with water. However, a large volume of research has focused on the application of Portland cement in mortar and concrete. For example, there exist thousands of publications regarding increasing the mechanical strength, workability, and chemical stability against the leaching chemicals of concrete and mortar [14,15]. However, no research was devoted to the application of Portland cement in membrane fabrication until 2014, when Wang et al. used cementitious membranes for ozone disinfection of water, according to our knowledge [16]. Recently, calcium aluminate cement, which is different than Portland cement, is also proved to be a promising material for membrane fabrication [17]. However, it would be better to avoid high-temperature sintering, where it is used for calcium aluminate membranes, for the sake of environmental protection [18].

One of the main challenges in membrane technology is the engineering of desired porosity, especially the fabrication of connected pores (channels), which will allow permeation without significant pressure resistance. Regarding this, the freeze-casting technique is a promising method for fabricating highly porous structures with connected pores and also, the feasibility of the method is already proven using cement and water or cement and tert-butyl alcohol systems [19,20]. Additionally, the freeze-casting technique is also considered as an eco-friendly method of porous body fabrication [21]. In freeze casting, the porosity of the materials is a template (replica) of solidified solvent initially contained in a slurry. In directional freeze-casting, water solidifies to lamellar-like ice crystals, which produce lamellar pores (channels) between ceramic walls after ice sublimation [21].

In this study, commercial ultrafine cement is used to fabricate membrane supports by the environmentally-friendly water-based freeze-casting technique. Recently, the freeze-casting technique has been applied to prepare cement membrane from ordinary Portland cement with a median particle size of either 5.1 µm or 7.65 µm while using toxic and expensive organic tert-butyl alcohol as a solvent and wet ball milling for a very long time (i.e., for 12–24 h) to obtain a homogenous slurry for freeze-casting [19,20]. However, this long wet-milling time of cement is energy consuming and it might influence the hydration process of the cement and, in consequence, the mechanical properties of the membrane [6]. The use of cement particle with a smaller size (i.e., <5 µm) might reduce the wet-milling time in order to obtain stable slurry for the freeze-casting process. Therefore, in this work, an ultrafine Portland cement with a d_95_ of 9.5 µm is applied, instead of ordinary Portland cement, which has d_95_ of 30–60 µm in general [22]. Using smaller particle sizes allows for overcoming the settling problem of cement slurry as well as short time (i.e., 15 min) of ball milling for slurry homogenization. In addition, the influence of solid content and freezing rate on the porosity, mechanical strength, and pore structure of the obtained membrane supports are also studied.

## 2. Materials and Methods 

### 2.1. Materials

Ultrafine Portland cement (U-PC) MIKRODUR^®^ P-U (d_95_ = 9.5 µm) was provided by Dyckerhoff GmbH (Wiesbaden, Germany). Polycarboxylatether based superplasticizer (SP) was supplied by MC-Bauchemie Müller GmbH & Co. KG (Bottrop, Germany) and then used as a dispersing agent for slurry preparation. Deionized water was used in all experiments. 

### 2.2. Fabrication of Membrane Supports

**Figure 1:**
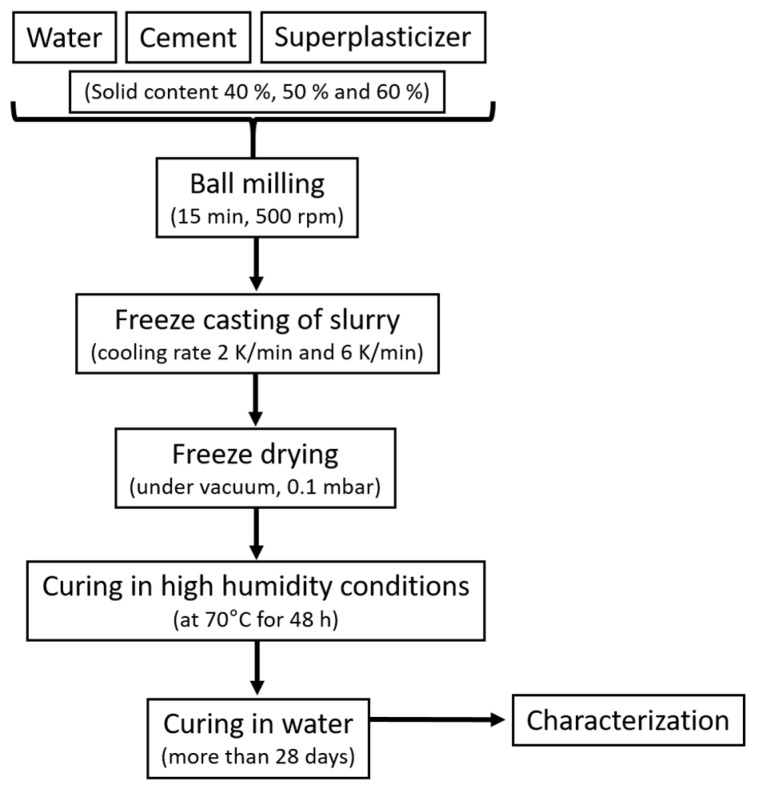
Schematic of the fabrication of cement membrane supports.

The slurry for freeze casting was prepared, as follows: U-PC, deionized water, and SP were mixed manually. Subsequently, the mixture was ball milled for 15 min with 500 rpm to obtain a homogeneous mixture of well-dispersed cement particles with the help of SP. The obtained suspension was poured into a PTFE mold (25 mm diameter and 4 mm height), which stood on a cold copper finger immersed into liquid nitrogen for freezing. The frozen samples were left to dry in the freeze dryer for 24 h. The dried samples were placed in a closed container containing water that is not in direct contact with the cement to generate moisture. The container is then heated at 70 °C in the oven for 48 h for curing the cement by humidity. After humid curing, the cement samples are strong enough to be removed from the mold and then immersed in water to complete hydration of the cement. The samples remain in the water until characterization, i.e., for more than 28 days. Before beginning the characterization, the samples were carefully polished from the bottom and topsides to obtain ~3 mm thick membrane supports with flat and smooth surfaces. Figure 1 shows the entire process scheme. Table 1 lists the names of the samples, solid content of the initial slurry, and freezing rates.

### 2.3. Characterization Methods

The open porosity of samples was measured by the Archimedes method, according to the ASTM C-373-18 [23]. The microstructure of samples was investigated by Scanning Electron Microscopy in an LEO 1530 (Carl Zeiss, Jena, Germany). The specimens for SEM characterization were cut from the prepared membrane using a diamond disc, and then polished and sputtered with a gold layer. The micro-computed tomographic (µCT) images were obtained using polychromatic radiation (pink beam) at the beamline for TOmographic Microscopy and Coherent rAdiology experimenTs (TOMCAT) at the Swiss Light Source of the Paul Scherrer Institut, Villigen, Switzerland. 1000 projections were taken within 10 s with a high-numerical-aperture macroscope, offering a resulting voxel size of 2.75 µm [24]. The images were then obtained by single distance phase retrieval [25] and reconstruction while using the gridrec algorithm [26]. The obtained volumetric data was processed using Avizo (Thermo Fisher Scientific, Waltham, MA, USA). Pore size distribution was analyzed with a mercury intrusion porosimeter (MIP) 2000 WS (Carlo Erba, Rodano, Italy).

The mechanical strength of disc-shaped, i.e., 25 mm diameter and 3 mm thick, membrane supports were characterized by the ring-on-ring biaxial flexural strength test (modified ASTM C1499—15 test). The support ring has a diameter of 22 mm and the loading ring has a diameter of 12 mm. The test was performed by a RetroLine testing machine with a testXpert v. 11 software (Zwick/Roell, Ulm, Germany). Details of the ring-on-ring fracture tests, along with the equations used for evaluation, have been reported elsewhere [27,28].

Pure water permeability of supports was tested by POROLUX 1000 (Porometer, Borgloon, Belgium), which comprises of the sample holder, gas-liquid exchanger, scale (Precisa ER 8200 C), and porometer Porolux 1000. The gas-liquid exchanger is filled with liquid, and it is connected to the Porolux 1000 at the pressure side. At the liquid side, the external sample holder is connected to the gas–liquid exchanger, and the drain tube from the sample holder is led into a glass beaker on a scale. The Porolux 1000 increases the pressure to a predefined target value and maintains this pressure for a predefined time period. The gas displaces the liquid in the gas–liquid exchanger, and the liquid is pressed towards the external sample holder until it will eventually flow through the sample. The filtration area of membranes was 2.986 cm^2^, the thickness was 3.2(±0.1) mm, and the duration of the test was 120 seconds.

## 3. Results and Discussion

### 3.1. Microstructure 

**Figure 2:**
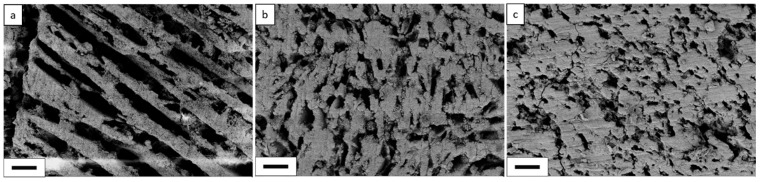
SEM images of specimens cross-sections perpendicular to freezing direction, frozen with 2 K/min cooling rate and different solid content: (**a**) 40 wt%; (**b**) 50 wt%; (**c**) 60 wt% (the scale bar is 100 µm).

In the first step, SEM and micro-computed tomographic (µCT) characterizations investigate the influence of solid content and the freezing rate on the open porosity and the microstructure of membrane supports. As found, solid content is one of the main factors that affect the microstructure of freeze cast samples. With increasing the solid content of initial slurry from 40 wt% to 60 wt%, the surface morphology and the pore structure of the samples changed significantly even with using the same cooling rate (i.e., 2 K/min), as it can be seen from SEM micrographs (Figure 2). Lamellar pore channels can be seen for the sample prepared with 40 wt% of solid content (Figure 2), which are parallel to the freezing direction of the solvent. This result is in good agreement with previous studies, which showed that water molecules are first frozen as small hexagonal ice nucleus and then grow to form lamellar ice crystals along the freezing direction during the freezing process [29,30]. Thus, lamellar pore channels are expected to be formed after sublimation. However, since pores are templates of those initial frozen liquid, i.e., porosity amount and their microstructure are directly proportional to the liquid content of slurry, type of liquid as well as the additives used [29,30]. The pore walls became thicker with increasing the solid content in the slurry, resulting in the change of pore geometry from lamellar to cellular, as can be seen in Figure 2. The microcracks that were observed on the surface of the samples could have resulted from the cutting and polishing processes used in the preparation of the specimens for SEM characterizations. 

**Figure 3:**
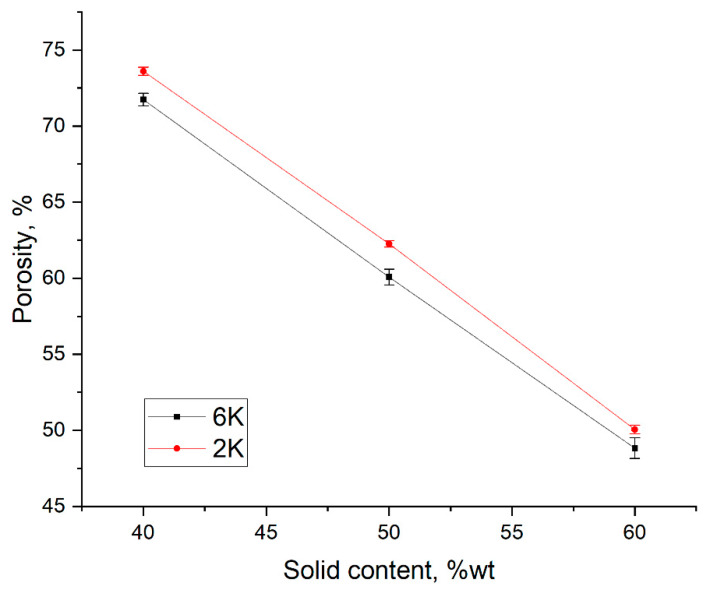
Influence of solid content on open porosity in specimens frozen at two different freezing rates.

**Figure 4:**
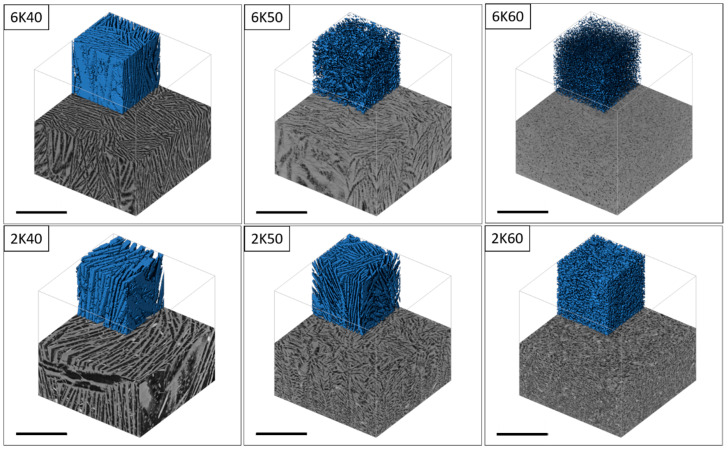
Micro-computed tomographic (µCT) horizontal cross-sections obtained perpendicular to freezing direction in freeze cast samples with different initial solid content (40 wt%, 50 wt%, and 60 wt%) frozen at 2 and 6 K/min (the scale bar is 1000 µm).

**Figure 5:**
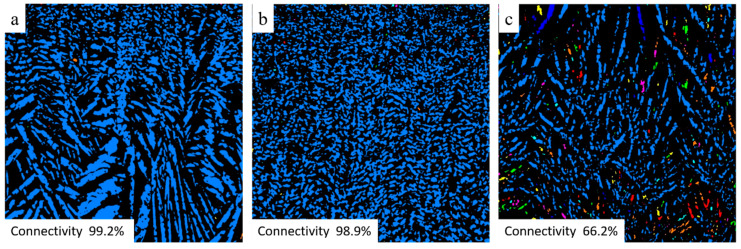
µCT images of the cross-section of 2K50 (**a**), 2K60 (**b**), and 6K50 (**c**) samples parallel to freezing direction. Connected pores touching the upper and lower sample boundary are displayed in blue, other colors represent trapped pores.

The open porosity in freeze cast samples varies almost linearly with solid content in the slurry regardless of the freezing rate, as shown in Figure 3. For example, in the specimens, frozen at 2 K/min, increasing solid content from 40 to 60 wt% results in a decrease in the open porosity from 73 % to 50%, respectively. When solid content in the slurry is low (i.e., 40 wt%), the growing ice crystals can easily repel cement particles and produce long ice crystals or lamellae, which becomes connected lamellar channels after ice sublimation. However, with increasing the solid content in the slurry to > 50 wt%, fewer ice crystals will grow between the solid particles; consequently, solid particles will easily restrain the growth of ice crystals rather than rearrange in front of the growing ice crystals. At high solid content, i.e., 60 wt%, despite porosity of around 50%, the connectivity of channels is nearly lost or very low, i.e., the lamellar structure changed to cellular, as can be observed from the SEM (Figure 2) and µCT (Figure 4 and Figure 5) images.

**Figure 6:**
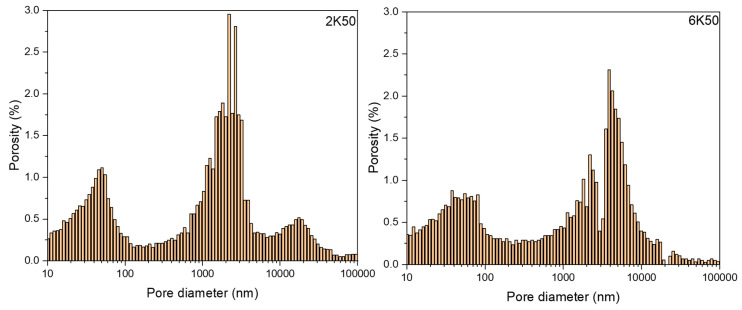
Pore size distribution in 2K50 and 6K50 samples.

The freezing rate does not have a large effect on the open porosity in materials. For example, 2K50 and 6K50 samples with the same solid content but frozen at different freezing rates display almost the same open porosity (Figure 3). However, they differ significantly in pore morphology (see µCT images in Figure 4) as well as in pore size distribution (Figure 6). Samples that were frozen at 2 K/min exhibit wider channels and a higher fraction of larger pores when compared to the samples that were frozen at 6 K/min. That means that the freezing rate is the main factor that defines the pore morphology and size. Higher freezing rate (solidification speed) of the slurry results in the formation of finer pores (cellular pore morphology) with lower channel connectivity. In agreement with previous studies [31,32], by increasing the freezing rate, more particles will be entrapped, resulting in smaller pores and lower connectivity. Ice crystals push away particles rather than trapping them at slower freezing rates.

For membrane applications, the connectivity of pores plays a vital role; thus, it is very important to choose the proper amount of solid content or freezing rate to maintain the connectivity of the pore channels. If high solid content is indispensable, then slow freezing (i.e., 2 K/min) should be applied to keep the connectivity of pores suitable for membrane applications [21,33].

### 3.2. Mechanical Stability of Membranes

The suitable mechanical stability of membranes, particularly water filtration membranes, is a critical factor for applications because membranes operate under transmembrane pressure differences ranging from 0.5 bar up to 2.0 bar. Therefore, in the next step, the flexural strength of the obtained membranes was measured while using a ring-on-ring biaxial flexural strength test. The ring-on-ring test has been applied in this work because it gives correct results for our disc-shaped freeze cast samples. For 3- or 4-point bending tests, the samples have to be prepared in a bar shape either by cutting freeze cast samples or freeze-casting the samples into the needed shape. Thus, both 3- and 4-point bending tests give inappropriate results, because of the additional stress/cracks formed in the tested samples from the cutting process. Moreover, the mechanical properties of such bar-shaped samples may not represent the whole body, as it depends strongly on the position, which, in turn, is characterized by different pore size distribution and morphology [34]. 

**Figure 7:**
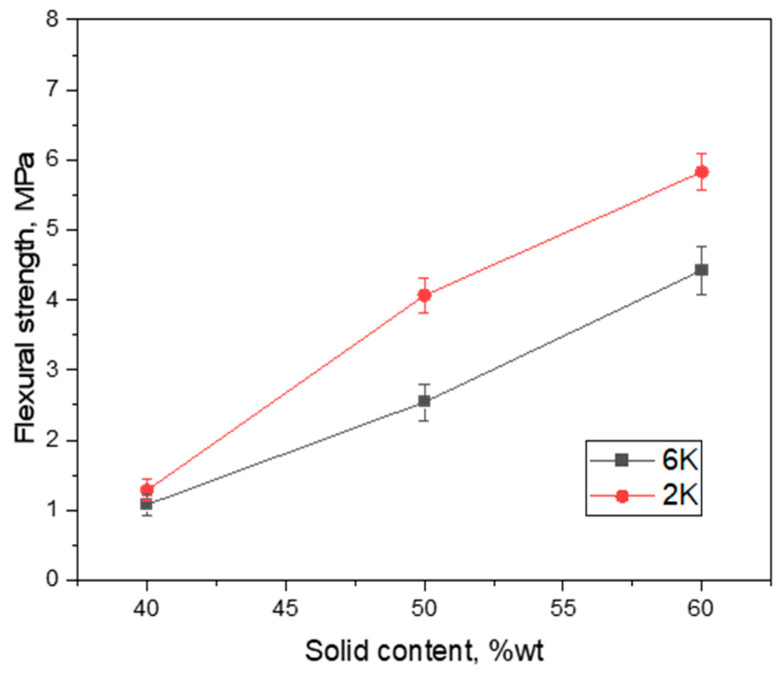
Influence of solid content and freezing rate on flexural strength of freeze cast cement samples.

As shown in Figure 7, the flexural strength of cement membrane support prepared in this work varies between 1.1 and 5.8 MPa depending on the solid content of initial slurry as well as the freezing conditions. This variation can be explained by taking into account the open porosity of the specimens as well as the pore size distribution and morphology. For example, the 2K40 and 6K40 samples with a quite similar pore fraction and morphology (see Figure 3 and Figure 4) also display very similar flexural strengths of 1.3 and 1.1 MPa, respectively. However, the situation is different for other samples with the same solid content, i.e., 2K50/6K50, as well as 2K60/6K60, which displays a slightly different flexural strength of 4.1/2.5 MPa and of 5.8/4.4 MPa, respectively. This difference might be due to the difference in the pore size distributions and morphology, i.e., samples that are frozen with low freezing rate, 2K50 and 2K60, have larger pores as compared to samples frozen with higher freezing rate, 6K50 and 6K60. Therefore, it can be concluded that lamellar pore morphology and larger pores are beneficial for the mechanical stability of ice-templated cement membranes.

The obtained flexural strength values for cement membrane supports that were prepared in this work are in good agreement with previously reported values of similar cement membrane supports prepared by freeze casting, see Table 2 [12,19,20]. However, the mechanical properties of membranes obtained from different sources can differ significantly because of different testing methods applied as well as sample features (such as the size of specimens, its position in the membrane, pore morphology, and directionality) [35]. Generally, the mechanical stability of all cement membranes, which are obtained by a sintering-free route, is low if compared with other low-cost ceramic membranes prepared by applying high-temperature sintering, as can be seen from Table 2. For instance, cement membranes exhibited compressive strengths up to 17 MPa, while fly ash and alumina mixture membranes demonstrated compressive strength of up to 45 MPa, both have similar porosities [12,36]. Nevertheless, the cement membrane supports prepared in this work using an initial solid loading of 50 and 60 wt% exhibit acceptable flexural strengths and are stable enough to be applied for water filtration tests. Besides this, when sintering-free route and higher porosity are also considered, the cement membranes fabricated in this work have quite good flexural strength as compared to other high-temperature sintered low-cost ceramic membranes with lower porosity, see Table 2.

Additionally, the mechanical strength of cement membrane supports could be further improved, for example, by reinforcing them with fibers or by blending reactive silica to the cement. The latter will react with calcium hydroxide by-product of cement hydration reaction in order to produce calcium silicate hydrate [46].

### 3.3. Permeability Performance 

Only 2K50, 2K60, and 6K50 samples were tested for water permeability because the samples frozen with low solid content (2K40 and 6K40) are very brittle and extremely sensitive to any mechanical stress. They also have a rougher, porous surface, and they would not withstand the pressure. Sample 6K60 has not also been tested, because it has almost no connected pores or very small pores, as shown in Figure 4, so no water permeation (at least very low) is expected. Measurements were done at 0.5 bar, and the results are presented in Table 3.

Despite equal porosities, the 2K50 and 6K50 samples display different pure water fluxes of 180.5 L/(h·m^2^) and 79.9 L/(h·m^2^), respectively. This difference in water fluxes of 2K50 and 6K50 samples can be explained by a higher amount of large pores in 2K50 in comparison with that in 6K50 sample, as revealed from µCT investigations (see Table 3). Surprisingly, 2K60 sample showed better water flux (i.e., 134.9 L/(h·m^2^)) than 6K50 sample (i.e., 79.9 L/(h·m^2^)), although the former has lower total porosity than latter. This result can be explained by the difference in the pore size distribution of both samples. Despite lower total porosity, the 2K60 sample contains a high amount of pores larger than 5 µm (i.e., 31 %), while, 6K60 sample has only 14% of its pores larger than 5 µm. Another reason could be the fraction of pores connected parallel to freezing direction, as it can be seen from µCT images, Figure 5, which is a cross-section of 2K50, 2K60, and 6K50 samples parallel to freezing direction. The analysis showed that the fraction of connected pores touching the upper and lower sample boundary (light blue areas) in the 2K50, 2K60, and 6K50 samples is 99.2%, 98.9%, and 66.2%, respectively (other colors represent trapped pores). This relatively low fraction of connected pores for sample 6K50 led to a low water permeation ability when compared to the 2K60 sample. 

Consequently, we can say that water permeability and porosity of samples, by Archimedes method and µCT investigations, showed that the permeation ability of water is not directly related to the porosity alone. The pore size and connectivity of pores are also very important. When large and connected pore exists, then water will be more easily permeated from those pores as compared to small pores.

The permeation ability of freeze-casted cement membranes frozen at 2 K/min in this work, e.g., 2K50, is much better than that previously reported in the literature, as shown in Table 2. This sample has pure water flux of 180.5 L/(h·m^2^), where 100 L/(h·m^2^) was reported for cement membrane in previous work [20] at the same transmembrane pressure of 0.5 bar. However, the porosity of samples is similar, i.e., 62% and 64%, respectively. This difference might result from the microstructure of pores, which is dependent on the solvent used and freezing conditions. We used water as a freezing solvent, but Dong et al. [20] used tert-butyl alcohol.

## 4. Conclusions

In this work, cement-based membrane supports were successfully fabricated from ultrafine Portland cement, which is a low-cost material and it develops mechanical strength by reacting with water without any need for high-temperature sintering. The application of the nature-inspired freeze casting method produced highly porous supports, which have connected channels along the freezing direction. While using different industry-relevant and scalable freezing rates, promising results could be obtained, particularly for the slurries containing 50% solid material. The freeze-casted cement membrane from this solid content exhibited high porosities of around 60% and comparable water flux of 180 L/(h·m^2^) and 80 L/(h·m^2^), with 2 K/min and 6 K/min freezing rate, respectively, while maintaining adequate mechanical strength. Moreover, additives and fibers can be used to increase the flexural strength of freeze cast cement membranes, which requires more research on this topic.

## Figures and Tables

**Table 1:** Overview of the specimens characterized in this study.

Sample	Solid Content of Slurry *, wt%	Cement, g	Water, g	Superplasticizer, g	Freezing Rate, K/min
2K40	40	4.0	6.0	0.04	2
2K50	50	5.0	5.0	0.1	2
2K60	60	6.0	4.0	0.12	2
6K40	40	4.0	6.0	0.04	6
6K50	50	5.0	5.0	0.1	6
6K60	60	6.0	4.0	0.12	6

* Solid content is shown without taking into account the amount of superplasticizer.

**Table 2:** Porosity and mechanical stability of low-cost membrane supports.

Material	Preparation Method	Sintering Temperature, °C	Porosity, %	Mechanical Stability, MPa*	Reference
Cement	Freeze casting	No sintering	48–73	1.3–5.8 (F)	This work
Cement	Freeze casting	No sintering	48–62	10.7–16.6 (C)	[12]
Cement	Freeze casting	No sintering	57–69	9.7–15.2 (C)	[20]
Cement	Freeze casting	No sintering	50–58	1.1–12.5 (C)	[19]
Fly ash + alumina	Freeze casting	1600	62	45 (C)	[36]
Mullite	Freeze casting	1550	66–77	23–33 (C)	[37]
Fly ash	Paste casting	800–1000	35–40	8–20 (F)	[38]
Kaolin + additives	Paste casting	850–1000	33–42	3-8 (F)	[39]
Kaolin	Extrusion	1000–1250	46–60	4–24 (F)	[40]
Apatite	Extrusion	1150	40	15 (F)	[41]
Kaolin + Dolomite	Pressing	1375	42–55	8–18 (F)	[42]
Moroccan Clay	Pressing	950	40	14 (F)	[43]
Natural zeolite	Pressing	800-900	13–38	4.5–6.0 (F)	[44]
Quartz sand	Pressing	850	23–35	15–39 (C)	[45]

* Type of mechanical testing method: F—bending (flexural) test; C—compressive test.

**Table 3:** Test conditions and results of water permeation analysis for freeze cast cement membrane supports.

Sample	Trans-Membrane Pressure, bar	Pure Water Flux, L/(h·m^2^)	Porosity by Archimedes Method (open porosity), %	Porosity by µCT (pores > 5 µm), %	Fraction of Connected Pores by µCT, %
6K50	0.5	80 ± 5	60.1 ± 0.5	14	66.2
2K50	0.5	181 ± 7	62.3 ± 0.2	35	99.2
2K60	0.5	135 ± 5	50.1 ± 0.3	31	98.9
